# Examining the Psychometric Properties and Factor Structure of EASE

**DOI:** 10.1093/geroni/igaf122.621

**Published:** 2025-12-31

**Authors:** Adam Davey, Margaret Calkins, Migette Kaup, Robert Wrublowsky

**Affiliations:** University of Delaware, Newark, Delaware, United States; IDEAS Institute, Cleveland Heights, Ohio, United States; Kansas State University, Manhattan, Kansas, United States; RAW Consulting, Winnipeg, Manitoba, Canada

## Abstract

Initial information about psychometric properties of EASE subscales as well as for the overall instrument will be discussed. Development of the tool has included initial determination of face-validity, followed by establishing construct validity (r = 0.88 with EAT tool), and interrater agreement (r = 0.97). With the administration of the EASE tool in 228 living areas (e.g., units), parallel analysis was used to investigate the structure of the 130 item EASE instrument. 12 components were initially identified, and several additional component analyses were examined to determine the most efficient structure. Results describe the structure of the EASE instrument with regard to living area characteristics, particularly focusing on items that discriminate between different models (traditional, household and hybrid). Recognition of the dual purposes of the EASE tool as both a design planning resource and as a tool for comparative research across multiple settings, different potential structures will be discussed.

